
JOURNAL INFORMATION
==============================
NLM Title Abbreviation: Tex Med J (Austin)
NLM Journal ID: 21130590R

Texas Medical Journal

ISSN: 0892-8495
Publisher: Biomedical Journal Digitization Project

ARTICLE INFORMATION
==============================
PMCID: PMC9615867
Article version: 1
Subjects: Books and Magazines

Diseases of the Digestive Organs in Infancy and Childhood, with Chapters on the Diet and General Management of Children, and Massage in Pediatrics

Print publication date: 1902 Mar
Volume: 17
Issue: 9
First page: 351
Last page: 351
License: This work is free of known copyright restrictions. For more information, please see PMC Back Issue Digitization.
License URL: https://creativecommons.org/publicdomain/mark/1.0/

==============================

Phillips' Emulsion 50% best NORWAY COD LIVER OIL minutely sub-divided, WITH WHEAT PHOSPHATES (Phillips')

Pancreatized, Palatable, Permanent, Miscible in Water, Milk, Wine, etc.

Phillips' Milk of Magnesia Mg H2 O2 (FLUID.) "THE PERFECT ANTACID."

for correcting Hyperacid conditions—local or systemic.
Vehicle for Salicylates, Iodides, Balsams, etc.

Phillips' Phospho-Muriate of Quinine, COMP.

TONIC AND RECONSTRUCTIVE.

WHEAT PHOSPHATES, WITH MURIATE OF QUININE AND STRYCHNINE.

PHILLIPS' WHEAT PHOSPHATES (ACID).
PHILLIPS' SYRUP OF WHEAT PHOSPHATES.
PHILLIPS' DIGESTIBLE COCOA.

THE CHAS. H. PHILLIPS CHEMICAL CO., NEW YORK

---

Diseases of the Digestive Organs in Infancy and Childhood, with chapters on the Diet and General Management of Children, and Massage in Pediatrics. By Louis Starr, M. D., late Clinical Professor of Diseases of Children in the Hospital of the University of Pennslyvania; Consulting Pediatrist to the Maternity Hospital, Philadelphia, etc. Third edition, rewritten and enlarged. Illustrated. Published by P. Blakiston's Son & Co., 1012 Walnut St., Philadelphia. 1901. Price, $3.00 net.

It has been ten years since the last edition of this book was published, and much revision and rewriting became necessary in the present edition. Several new chapters have been added, and several obsolete ones left out. Among the new chapters are Simple Atrophy, Infantile Scurvy, Rickets, Lithemia, Infectious Follicular Tonsillitis, Naso-Pharyngeal Adenoid, Hypertrophy, Proctitis and Apendicitis. The section on feeding has been most carefully revised, and great stress has been laid upon hygienic measures.

Starr's is a popular book, and in no respect is it extravagantly written. It is concise and to the point. There is but one criticism the reviewer can make, and that is the lack of illustrations in the text. T. J. B.
